# The expression of diacylglycerol kinase theta during the organogenesis of mouse embryos

**DOI:** 10.1186/1471-213X-13-35

**Published:** 2013-10-01

**Authors:** Shuji Ueda, Becky Tu-Sekine, Minoru Yamanoue, Daniel M Raben, Yasuhito Shirai

**Affiliations:** 1 Department of Agrobioscience, Graduate School of Agricultural Science, Kobe University, Kobe, Hyogo, Japan; 2 Department of Biological Chemistry, Johns Hopkins University School of Medicine, Baltimore, Maryland, USA

**Keywords:** Diacylglycerol kinase, Embryonic development, Central nerve system, Epidermis

## Abstract

**Background:**

Diacylglycerol kinase (DGK) is a key enzyme that regulates diacylglycerol (DG) turnover and is involved in a variety of physiological functions. The isoform DGKθ has a unique domain structure and is the sole member of type V DGK. To reveal the spatial and temporal expression of DGKθ we performed immunohistochemical staining on paraffin sections of mouse embryos.

**Results:**

At an early stage of development (E10.5 and 11.5), the expression of DGKθ was prominently detected in the brain, spinal cord, dorsal root ganglion, and limb bud, and was also moderately detected in the bulbus cordis and the primordium of the liver and gut. At later stages (E12.5 and 14.5), DGKθ expression persisted or increased in the neocortex, epithalamus, hypothalamus, medulla oblongata, and pons. DGKθ was also evident in the epidermis, and nearly all epithelia of the oropharyngeal membrane, digestive tract, and bronchea. At prenatal developmental stages (E16.5 and E18.5), the expression pattern of DGKθ was maintained in the central nervous system, intestine, and kidney, but was attenuated in the differentiated epidermis.

**Conclusion:**

These results suggest that DGKθ may play important physiological roles not only in the brain, but also in diverse organs and tissues during the embryonic stages.

## Background

Many hormones and growth factors stimulate phospholipase C by activating its receptor. This activation results in the production of diacylglycerol (DG) at the plasma membrane, which triggers the activation of several enzymes, such as chimerins [1], Ras guanyl nucleotide-releasing protein (RasGRPs) [2,3], transient receptor potential cation channel C (TRPC) [4], and both conventional and novel PKCs [5,6]. DG kinase (DGK) phosphorylates DG to produce phosphatidic acid (PA), resulting in the inhibition of DG-mediated intracellular signal transduction. Additionally, PA is a lipid second messenger that regulates various target proteins, including atypical PKC, mTOR, and phosphatidylinositol 4-phosphate 5-kinase [7,8]. Thus, DGK plays pivotal roles in various intracellular signaling pathways by regulating the DG and PA levels [9-12]. Ten DGK isoforms have been identified in mammal, and classified into five subtypes, from I to V, based on similarities in the domain structures.

The physiologic roles of DGK isoforms have been partially elucidated by analyses of DGK gene expression patterns and of genetically modified organisms. For example, DGKβ is a major isoform expressed in the brain, and the disruption of this isoform impairs memory and causes antidepressant-like effects in mice [13]. In contrast, DGKα enhances interleukin 2-induced T cell proliferation [14], and knockout (KO) mice exhibit impaired induction of T cell anergy [15]. Another isoform, DGKζ is a type IV DGK that is ubiquitously expressed in most mouse tissues. DGK*ζ* KO mice exhibit abnormalities in multiple tissues, including a decrease in the number of dendritic spines, and an impairment of the immune response [4,16,17]. In regard to nervous system, a large number of studies have shown the roles of DGKs in neuronal spine density, synaptic activity, epileptogenesis and neuronal plasticity in mammals [18,19] and C. elegans used as a genetic model [20]. It is not clear the relationships of ten DGK isoforms in mammal. To better understand the role of each DGK and its redundancy among the DGK family, further studies in other subtypes of DGKs are necessary.

DGKθ was originally cloned from the rat brain and identified as the sole type V DGK [21]. DGKθ contains three C1 domains and a Ras-association (RA) domain in the central region. Studies have shown that DGKθ is enriched in the nuclear matrix of various cultured cell lines [22], is negatively regulated by its interaction with the small GTPase RhoA [23], and translocate to the plasma membrane following phosphorylation by PKCϵ [24]. The optimal activation of DGKθ may require both polybasic protein and acidic phospholipid cofactors, which have been shown to stimulate DGKθ synergistically *in vitro*[25,26]. These multiple regulatory mechanisms enable DGKθ to mediate signals from a wide variety of extracellular ligands [24,27] including epidermal growth factor [28], nerve growth factor [29], α-thrombin [30], and adenosine [31].

DGKθ is highly expressed in rat brain [21] and is present in a variety of cultured cell lines [22,24]. However, there is limited information regarding this enzyme’s cell type-specific functions or its physiological roles in mammals. In this study, we examined the distribution and changes in expression from E10 to E17 to reveal the spatial and temporal expression of DGKθ protein in mouse embryos.

## Results

### Expression patterns of DGKθ in E10.5 to E16.5 mouse embryos

To determine the distribution of DGKθ, we performed immunohistochemistry (IHC) on paraffin sections of mouse embryos. We used two individual antibodies that target the C-terminal region of the protein, anti-DGKθ antibodies #1 and #2, to validate immunostaining patterns. Both antibodies detected a major 110-kDa band of DGKθ on an immunoblotting membrane containing the extract from whole brain (Additional file 1: Figure S1 C), in which DGKθ had been detected with RT-PCR (data not shown). The observed molecular size coincided with the size of endogenous protein (110 kDa) in HEK293 and HeLa cell lysates by immunoblotting [16,23]. The specificity of this antibody was further confirmed by the transient transfection. These antibodies successfully detected exogenously expressed recombinant DGKθ protein by immunofluorescence in HeLa cells, but not other subtypes (Additional file 2: Figure S2 and data not shown).

**Figure 1:**
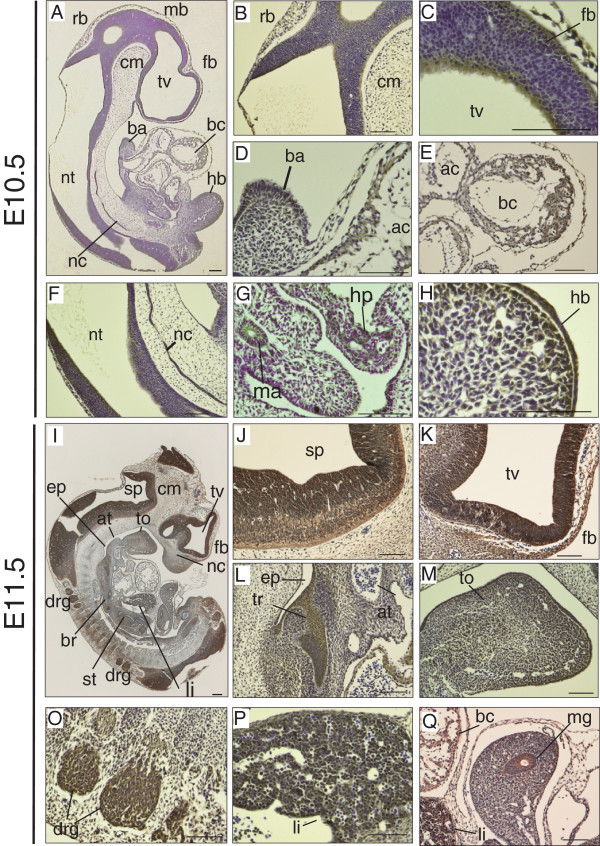
**Expression patterns of DGKθ at E10.5 and E11.5. (A, I)** Sagittal sections showing the distribution of DGKθ. **(B-H)** High magnification images showing DGKθ-positive regions at E10.5. Forebrain (fb), midbrain (mb), rhombencephalon (rb), third ventricle (tv), cephalic mesenchyme (cm), branchial arch (ba), atrial chamber (ac), bulbus cordis (bc), notochord (nc), hepatic primordium (hp), midgut artery (ma), hindlimb bud (hb). **(J-Q)** High magnification images showing DGKθ positive region at E11.5. Spinal cord (sp), esophagus (ep), nasal placode (np), trachea (tr), tongue (to), bronchi (br), stomach (st), liver (li), arterial trunk aortic arch (aa), dorsal root ganglion (drg), midgut (mg). The sections were stained with anti-DGKθ antibody #1. The scale bars show 100 μm.

At E10.5, the immunoreactivity of DGKθ was noticeable along the surface of the forebrain (fb), rhombencephalon (rb), and neural tube (nt) (Figure 1A-C and F), and was also distinctly observable in the ectodermal epithelium of the hind bud (hb) (Figure 1H) (evident as brown staining in all images). DGKθ expression was also detected in the branchial arch (ba), bulbus cordis (bc), hepatic primordium (hp), midgut artery (ma), and notal cord (nc) (Figure 1A). A similar staining pattern persisted at E11.5. Immunoreactivity of DGKθ was detected in the bulbus cordis, liver (li), and lumen of the stomach and midgut (mg) (Figure 1I and M). In addition to the distribution observed at E10.5, DGKθ staining was noted in the tongue (to), nasal placode (np), primitive bronchi (br), the dorsal root ganglion (drg), and the trachea (tr) separating from the esophagus at E11.5 (Figure 1 I, K and L). It is noteworthy that the immunoreactivity significantly increased in the neuroepithelium surrounding the third ventricle (tv) at E11.5 compared with that at E10.5 (Figure 1K). In contrast to the faint staining of the mesenchymal cells around the cephalic region, the immunoreactivity of the mesenchymal cells around the peripheries of the tongue (to) and nasal placode (np) was relatively high (Figure 1A-B and I).

**Figure 2:**
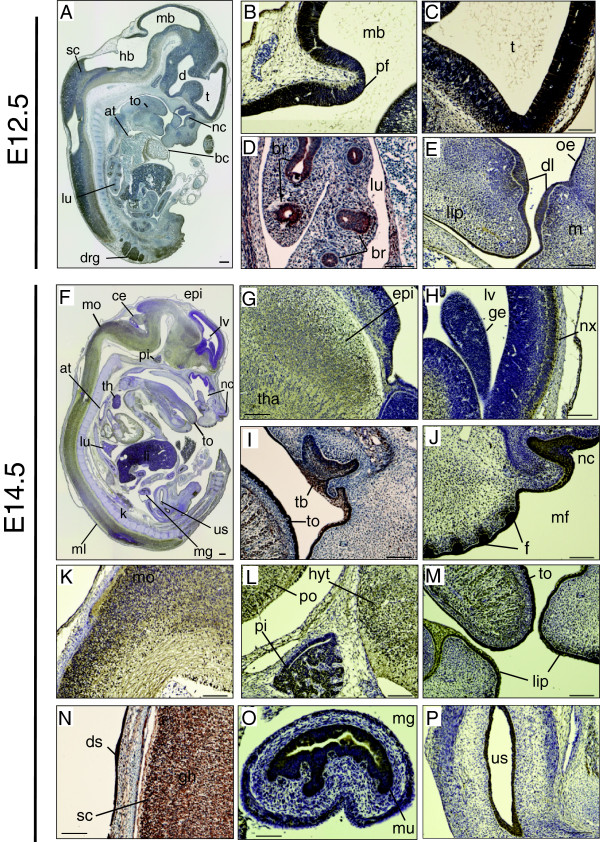
**Expression patterns of DGKθ at E12.5 and E14.5. (A, F)** Sagittal sections showing the distribution of DGKθ. **(B-E)** High-magnification images showing DGKθ-positive regions at E12.5. Telencephalon (t), diencephalon (d), midbrain (mb), arterial trunk (at), tongue (to), lung (lu), dorsal root ganglion (drg), bronchus (br), dental lamina (dl), oral epithelium (oe). **(F-P)** High-magnification images showing DGKθ-positive regions at E14.5. Neocortex (nx), lateral ventricle (lv), ganglionic eminence (ge), epithalamus (epi), cerebellum (ce), medulla oblongata (mo), pituitary gland (pi), pons (po), hypothalamus (hyt), nasal cavity (nc), tooth bud (tb), follicle (f), lung (lu), liver (li), dorsal skin (dr), midgut (mg), mucosa layer (mu), urogenital sinus (us), kidney (k). The sections were stained with anti-DGKθ antibody #1. The scale bars indicate 100 μm.

The embryonic brain grows rapidly until E12.5, and the forebrain partly divides into the telencephalon (t) and diencephalon (d). Simultaneously, a pharynx membrane and respiratory apparatus rapidly develop. In the current study, at E12.5 the immunoreactivity in the cephalic region increased intensively at the rostral side of the telencephalon, the tectum of midbrain, and the pontine flexure (pf) in which neuroepithelial cells proliferate (Figure 2A-C). Furthermore, the immunoreactivity persisted or increased in multiple organs (tongue, lung, liver, heart, and midgut) (Figure 2A and D), and expanded to the surface layer of the nasal cavity and dental lamina (dl) (Figure 2E). A similar pattern of distribution was observed at E13.5 (data not shown).

In the developing central nervous system region at E14.5, DGKθ immunoreactivity was widely distributed in all brain regions. Particularly dense immunoreactivity was observed in the neocortex (nx), epithalamus (epi), medulla oblongata (mo), and the gray horn of spinal cord (Figure 2F-I, K and N). However, DGKθ was not detected in the ganglionic eminence (ge), which localizes to the medial side of the lateral ventricle (Figure 2H). In other areas, dense immunoreactivity was continually detected within various types of epithelia, including the tongue (to), tooth bud (tb), nasal cavity (nc), bronchus (br), midgut (mg), and urogenital sinus (us) (Figure 2F, I, J, M, O and P). In contrast to the immunoreactivity predominantly observed in the epithelia classified as simple or stratified epithelium, DGKθ protein was undetectable in the endothelial cells of the arterial trunk (Figure 2A and F). Beginning at E14.5, the fetal skin increased in thickness and formed the layer of the squamous epithelium. At the facial and dorsal surfaces we found a significant increase of immunoreactivity in the squamous epithelial cells, which successively differentiate into epidermis (Figure 2J and N). IHC without the DGKθ antibody failed to detect any signal in the sections from E10.5 to E14.5 (data not shown).

### Expression patterns of DGKθ in late stages of embryogenesis

**Figure 3:**
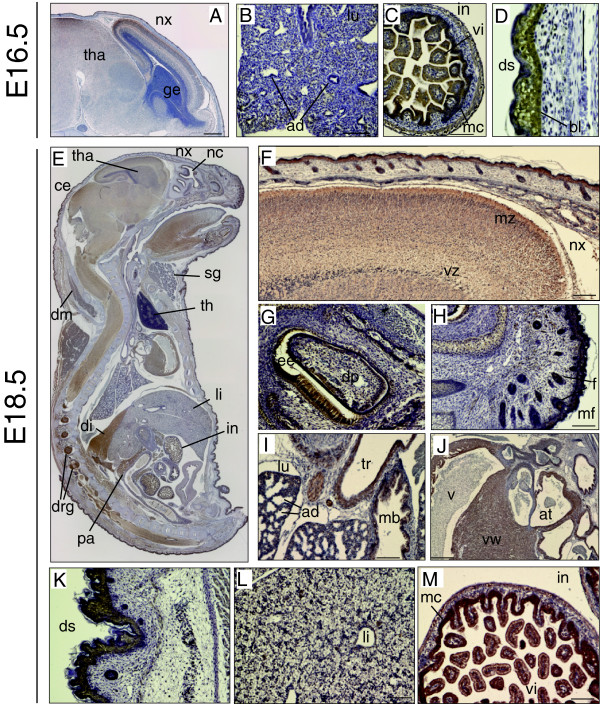
**Expression patterns of DGKθ at E16.5 and E18.5. (A, E)** Sagittal sections showing the distribution of DGKθ. **(B-D)** High-magnification images showing discriminative regions at E16.5. Thalamus (tha), neocortex (nx), ganglionic eminence (ge), lung (lu), alveolar duct (ad), intestine (in), villi (vi), mucosa (mc), dorsal skin (ds), basal layer (bl). **(E-M)** High magnification images showing discriminative regions at E18.5. cerebellum (ce), salivary glands (sg), thymus (th), dorsal muscles (dm), diaphragm (di), dorsal root ganglion (drg), pancreas (pa), marginal zone (mz), ventricular zone (vz), dental pulp (dp), enamel epithelium (ee), follicle (f), muffle (mf), throat (tr), aortic trunk (at), ventricle (v), ventricular wall (vw) The sections were stained with anti-DGKθ antibody #1. The scale bars indicate 100 μm.

At E16.5, the immunoreactivity of DGKθ was maintained in the neocortex (nx) and the mesencephalon (Figure 3A), and increased in the villi (vi) and mucosa (mu) of the intestine (Figure 3C) and the basal layer (bl) of the dorsal skin (ds) (Figure 3D). In contrast, we found that the immunoreactivity of DGKθ was attenuated in the lung (Figure 3B) and liver at E16.5. At prenatal developmental stages (E17.5 and E18.5), DGKθ immunoreactivity was unchanged in the brain region relative to the E14.5. In other areas, immunoreactivity was faintly observed in the enamel epithelium of incisor teeth (Figure 3E), the epidermis of the facial and dorsal surfaces (Figure 3H and K), the walls of the tranche and main bronchus, and the ventricular wall of the heart (Figure 3I and J), while strong signals persisted in the villi and mucosa of the intestine (Figure 3M) and in the collecting tubule (ct) and Bowman’s capsule (bc) of the kidney (Additional file 3: Figure S3 A and D). The intensity and pattern of the immunoreactivity was also verified by examining serial sections with two different anti-DGKθ (antibodies #1 and #2) (Additional file 3: Figure S3 B and C). When the anti-DGKθ antibody was preincubated with the blocking polypeptide prior to IHC, no immunostaining was detected at E17.5 with the exception of the pancreas (pa) and the surface of the villi in the duodenum, which corresponded with IHC results obtained without the primary antibody (Additional file 3: Figure S3 D and E).

### RT-PCR analysis of DGKθ expression in mouse embryos

**Figure 4:**
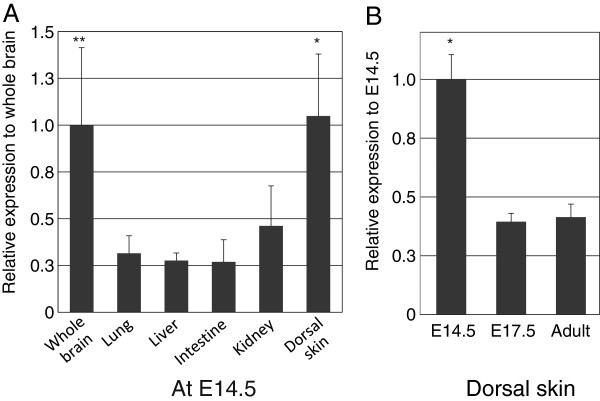
**RT-PCR analysis of DGKθ expression in mouse embryo.** The DGKθ mRNA levels were determined by semiquantitative RT-PCR. The number of amplification cycles for DGKθ and GAPDH were 35 and 28, respectively. The amount of template for each PCR was normalized to the expression level of GAPDH. **(A)** Relative expression of DGKθ in diverse organs at E14.5. The values (mean ± SEM, n = 4) represent the relative level of expression in the whole brain compared with the lung, liver, intestine, or kidney. ** indicates *P* < 0.01 and * indicates *P* < 0.05 **(B)** Relative expression of DGKθ at each developmental stage in the epidermis of the dorsal skin. The amount of template for each PCR was normalized to the expression level of GAPDH. The values (mean ± SEM, n = 4) represent the relative level of expression at E14.5. * indicates P < 0.05 compared with E17.5 or adulthood skin.

To confirm the distribution of DGKθ, we analyzed the expression of the DGKθ mRNA at E14.5 using semiquantitative RT-PCR. The results demonstrated that DGKθ was expressed in multiple organs (brain, lung, liver, intestine, kidney, and dorsal skin) at E14.5. Additionally, DGKθ was expressed more abundantly in the brain and dorsal skin than in the other organs (Figure 4A). This expression profile of the mRNA is consistent with the immunohistochemical observation, which indicated that DGKθ was distributed among various epithelia and neurons at E14.5.

To examine the change in DGKθ expression during development, we analyzed the expression in dorsal skin from E14.5 until adulthood. In the developmental skin, the expression of DGKθ was significantly higher at E14.5 than at E17.5 and during adulthood (Figure 4A and B).

## Discussion

To examine the regional distribution of DGKθ during the embryonic period, we performed immunohistochemistry (IHC) on whole mouse embryos. The immunostaining patterns indicated DGKθ protein was widely expressed in multiple organs and abundantly expressed in the brain and dorsal skin of mouse embryos. These results were confirmed by the analysis of DGKθ mRNA using RT-PCR and *in situ* hybridization (Figure 4 and Additional file 1: Figure S1B). The IHC results demonstrated that the expression of DGKθ significantly increased in the neuroepithelium surrounding the neural tube and ventricles in the brain. Prominent immunoreactivity of DGKθ was observed in a variety of neurons in the gestational brain over the period examined (E10.5-18.5). Especially, DGKθ was detected in the marginal zone of the neocortex, but not in the medial side of the lateral ventricle at E14.5 and E16.5. Since developing mammalian telencephalon is known to require atypical PKC [32], these results may suggest that DGKθ is associated with differentiation of the neuronal lineage or the locomotion of immature neurons [33]. During the prenatal period (E18.5), DGKθ was expressed in the developing cerebral cortex, hippocampus, and cerebellum. This distribution pattern is consistent with the *in situ* hybridization data for the adult rat brain [21]. Other DGK isoforms are known to be expressed in the postnatal brain [34-36] and are needed for various roles of mature neurons [37-39]. C.elegans DGK-1, which has 39% identity to mouse DGKθ, regulates DAG levels generated by heterotrimeric G protein signaling in response to the neurotransmitters in nematode [40]. Genetic analysis of C.elegans, dgk-1 shows the role of dopamine controlled locomotion and serotonin-controlled egg-laying behavior [41]. The presence of DGKθ throughout the prenatal brain at E18.5 suggests either that DGKθ is involved in a common neuronal process, or that it provides a level of redundancy for other DGK isoforms in neurons [29,39].

In the periphery, IHC results revealed that DGKθ is ubiquitously expressed in the layer of multiple organs during the embryonic period. In the intestine and kidney, the expression of DGKθ was prominent and persisted from E12.5 up to E18.5, while DGKθ expression in the lung, liver, and oropharyngeal membrane surrounding the tongue and nasal cavity was transient and attenuated before birth. Since DGKθ enhances the activation of EGF receptor stimulated with EGF via the counteraction of PKC activity in epidermoid cells [28], DGKθ may notably contribute to the development of epithelial cells during organogenesis (E10.5-E17.5). RT-PCR analysis demonstrated that the DGKθ mRNA was expressed in abundance throughout the developmental process, with highest expression in multiple key organs. Thus, DGKθ may have persistent roles in diverse organs and tissues during the embryonic stages.

## Conclusions

We revealed for the first time the distribution of DGKθ during the embryonic period. These results suggest that DGKθ may play important physiological roles not only in the brain, but also in diverse organs and tissues during the embryonic stages, and will serve as a basis for future in vivo genetic, functional, and mutational analyses of DGKθ.

## Methods

### Animals

Eight-week-old mice were purchased from Nippon SLC, Inc. (Hamamatsu, Japan). The animal works were reviewed and approved by the Care and Use of Laboratory Animals of Kobe University. All procedures using experimental animals were performed according to the Guidelines for the Care and Use of Laboratory Animals of Kobe University.

### Immunohistochemistry

Most chemical reagents were purchased from Wako Pure Chemical Inc. (Oosaka, Japan). C57BL/6 J mice were anesthetized and transcardially perfused with cold PBS(-) and fixed in 4% paraformaldehyde in PBS (-) for 10 min. Mouse embryos were isolated from pregnant mice and fixed in 4% paraformaldehyde in PBS (-) overnight [42]. The mouse embryos were paraffin-embedded and sectioned at a 5 μm thickness by Kyoudoubyouri, Inc. (Kobe, Japan) or Genostaff, Inc. (Tokyo, Japan). The serial paraffin sections were deparaffinized in xylene, and rehydrated using a graded series of ethanol from 99% to 50% and washed in distilled water. The sections were then incubated in 0.03% H_2_O_2_ in PBS (-) for 1 hr, and permeabilized with 0.3% Triton X -100 in PBS (-) for 30 min. Subsequently, the sections were washed with 0.1% Tween 20 in PBS (-), and incubated in 0.1% Tween 20 in PBS (-) supplemented with goat serum (Dako, Glostrup, Denmark), serving as blocking reagent, for 1 hr. Next, the sections were treated with an anti-DGKθ antibody diluted (antibody #1, 1:50 ; antibody #2, 1:100) in Can Get Signal immunostain solution A (Toyobo, Tokyo, Japan) overnight. The antibodies against DGKθ, antibody #1 (immunogen of 691–820 aa) and antibody #2 (immunogen of 677–883 aa), were purchased from Santa Cruz Biotechnology (California, USA), and BD Biosciences (California, USA), respectively. The control samples were probed with antibody that had been pre-incubated with a DGKθ blocking polypeptide. The bound antibodies were visualized using EnVision + System-HRP (Dako). The stained sections were dehydrated using a graded series of ethanol and mounted using a coverslip and Mount-Quick (Daido Sangyo, Gunma, Japan). The specimens were then photographed using an All-In-One Microscope system (BZ-8000 and BZ9000, Keyence, Japan), as reported previously [43].

The specific blocking polypeptide of DGKθ (677–883 aa) was generated by PCR using the following primers, with pEGFP-N3-DGKθ as template: 5′-AGTCGGATCCGGCACAGGGAATGACCTTG-3′ and 5′-CGAGTCACGCTCCTCAAGTGATGAGGATCCAGTG-3′. The amplified PCR fragment was subcloned into the pQE30 vector (Qiagen, Tokyo, Japan) for expression and purification from *E. coli*.

### Reverse transcription–PCR analysis

Reverse transcription-PCR (RT-PCR) was performed as described previously [44]. Briefly, organs or tissues were dissected from 15 embryos of mice under a stereomicroscope and frozen immediately on dry ice. The total RNA was isolated using a High Pure RNA Tissue Kit (Roche, Mannheim, Germany). For each tissue, equal amounts of RNA were reverse-transcribed with ReverTra Ace (Toyobo). The resulting cDNA was appropriately diluted and used to perform PCR with KOD-FX Neo (Toyobo).

The primer pair for mouse DGKθ was purchased from Takara-Bio Inc. (Otsu, Japan) and used to amplify the specific sequence : 5′-GGTTCAGAACAGGGCCAGGTAG-3′ and 5′-AGGGCTGTAGGCAGGCAAAC-3′ (NCBI : NM_199011, nt.3577-3706). RT-PCR was performed according to the manufacture′s specification. Reactions for DGKθ were performed for 35 cycles at 98°C for 10 s, 60°C for 30 s, and 68°C for 1 min. After electrophoretic separation, the amplified fragments were quantified using the image-analysis software Image J.

## Competing interests

All authors of this paper declare no conflict of interest.

## Authors’ contributions

SU designed the experiments and analyzed the data. SU wrote the manuscript. BT provided good comments and helped to write the manuscript. All authors read and approved the final manuscript.

## Supplementary Material

Additional file 1: Figure S1 Conformation of DGKθ expression patterns by *in situ* hybridization and western blotting. (A) High-magnification images showing the cardiac end of the stomach, and dorsal skin at E17.5. Epithelium (ep), dorsal skin (ds). (B) Sagittal sections showing the DGKθ mRNA expression by *in situ* hybridization analysis at E17.5. *In situ* hybridization were subjected to general method with digoxigenin (DIG)-labeled RNA antisense probe and visualized by anti-DIG antibody conjugated with alkaline phosphatase and the substrate NBT/BCIP (Roche). The specific probe (541–967 bp: the length 427 bp) were generated by DIG RNA Labeling kit [T7 RNA polymerase] using purified mouse DGKθ PCR product (541–967 bp: the length 427 bp) which produced by the following primers and mouse whole brain cDNA library : 5’-AGGGAGGGGAACCTGCCTTC-3’ and 5’-GATCGAATTCTAATACGACTCACTATAGGGCCTCATTCCGAGCCAGGCGGG-3’. The deparaffinized sections were incubated for 16 hr at 42°C in 4 × SSC containing 40% formamide, 0.1 × Denhardt’s solution, 10 mM DTT, and DIG-labeled RNA antisense probe (1 μg/ml). After hybridization, the sections were washed twice for 15 min at 37°C with 2 × SSC and subsequently washed twice for 5 min at 37°C with 0.4 × SSC. (C) Estimation of the specificity of the anti-DGKθ antibodies. Adult mouse heart and whole brain were lysed in RIPA buffer containing protease inhibitor cocktails (Nacalai tesque). Total cell lysate (30 μg protein) was applied to SDS-polyacrylamide gel electrophoresis (7.5%) and subjected to western blotting with anti-DGKθ antibody diluted (antibody #1, 1:100; antibody #2, 1:200) in Can Get Signal solution (Toyobo). The numbers on the right margin represent the molecular sizes of the pre-stained protein standard. Click here for file

Additional file 2: Figure S2 Specificity of the anti-DGKθ antibody against DGKθ and other DGKs. EGFP-fused DGKθ [45] and DGKβ [13] were transiently expressed in HeLa cells and cultured for 24 hr. The cells were fixed with 4% paraformaldehyde in PBS (-) and permeabilized with 0.3% Triton X -100 in PBS (-). Immunofluorescence staining was performed using the indicated antibodies followed by usual method [46]. Scale bar, 20 μm. Click here for file

Additional file 3: Figure S3 Expression patterns of DGKθ at E17.5. (A) High-magnification images showing the area of the renal medulla at E17.5. Collecting tubule (ct), Bowman’s capsule (bc). (B-H) Sagittal sections showing the staining pattern of anti-DGKθ antibody in abdomen and head area. Stomach (st), pancreas (pa), kidney (ki), duodenum (du), liver (li), neocortex (nx). Click here for file
