# Supplementary figures and images for: The expression of diacylglycerol kinase theta during the organogenesis of mouse embryos
Source: BMC Dev Biol. 2013 Oct 1;13:35. doi: 10.1186/1471-213X-13-35 (PMC3850696; doi:10.1186/1471-213X-13-35)

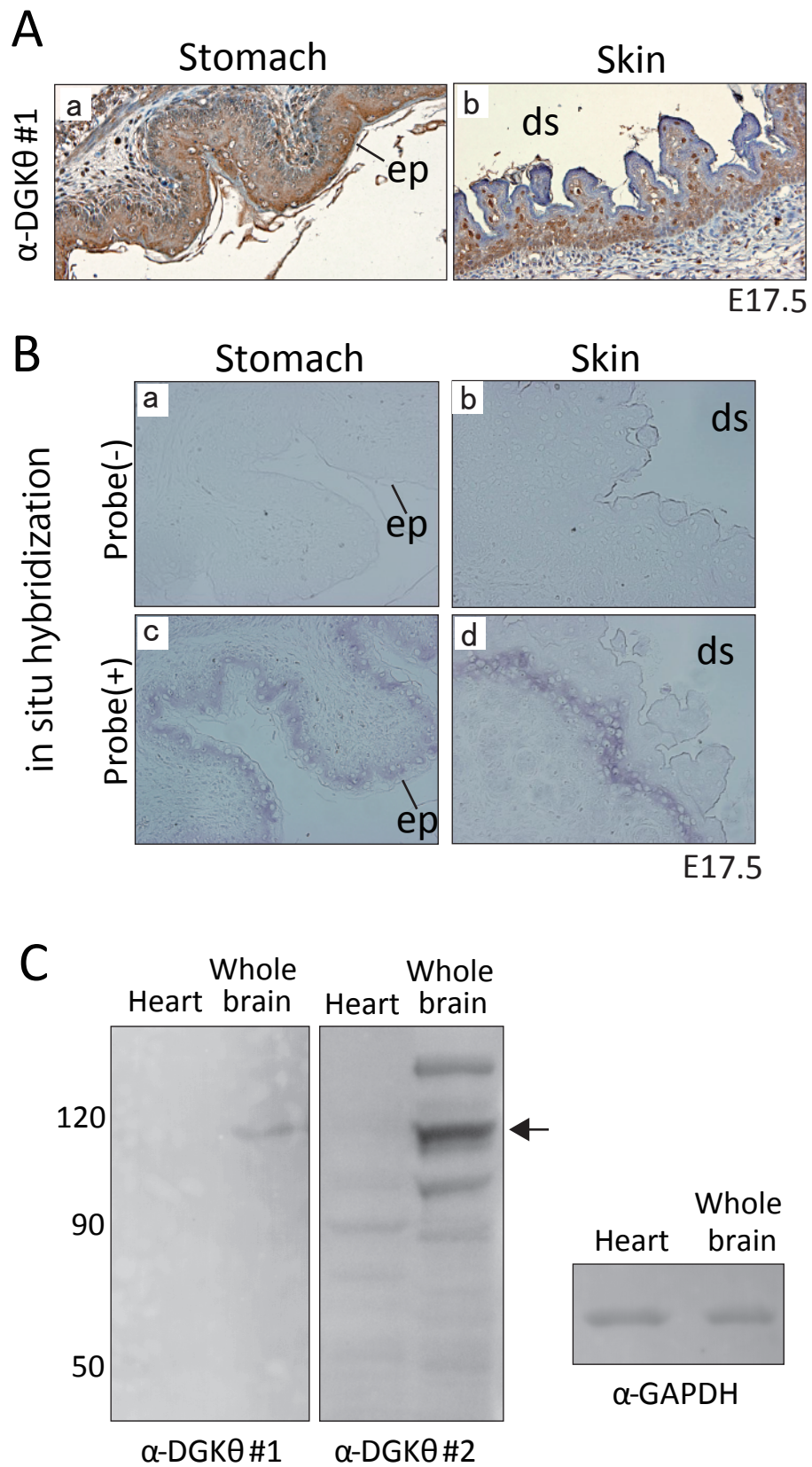

Figure S3. Ueda et al.

Supplement: Additional file 1: Figure S1 — Conformation of DGKθ expression patterns by in situ hybridization and western blotting. (A) High-magnification images showing the cardiac end of the stomach, and dorsal skin at E17.5. Epithelium (ep), dorsal skin (ds). (B) Sagittal sections showing the DGKθ mRNA expression by in situ hybridization analysis at E17.5. In situ hybridization were subjected to general method with digoxigenin (DIG)-labeled RNA antisense probe and visualized by anti-DIG antibody conjugated with alkaline phosphatase and the substrate NBT/BCIP (Roche). The specific probe (541–967 bp: the length 427 bp) were generated by DIG RNA Labeling kit [T7 RNA polymerase] using purified mouse DGKθ PCR product (541–967 bp: the length 427 bp) which produced by the following primers and mouse whole brain cDNA library : 5’-AGGGAGGGGAACCTGCCTTC-3’ and 5’-GATCGAATTCTAATACGACTCACTATAGGGCCTCATTCCGAGCCAGGCGGG-3’. The deparaffinized sections were incubated for 16 hr at 42°C in 4 × SSC containing 40% formamide, 0.1 × Denhardt’s solution, 10 mM DTT, and DIG-labeled RNA antisense probe (1 μg/ml). After hybridization, the sections were washed twice for 15 min at 37°C with 2 × SSC and subsequently washed twice for 5 min at 37°C with 0.4 × SSC. (C) Estimation of the specificity of the anti-DGKθ antibodies. Adult mouse heart and whole brain were lysed in RIPA buffer containing protease inhibitor cocktails (Nacalai tesque). Total cell lysate (30 μg protein) was applied to SDS-polyacrylamide gel electrophoresis (7.5%) and subjected to western blotting with anti-DGKθ antibody diluted (antibody #1, 1:100; antibody #2, 1:200) in Can Get Signal solution (Toyobo). The numbers on the right margin represent the molecular sizes of the pre-stained protein standard. [file 1471-213X-13-35-S1.pdf]

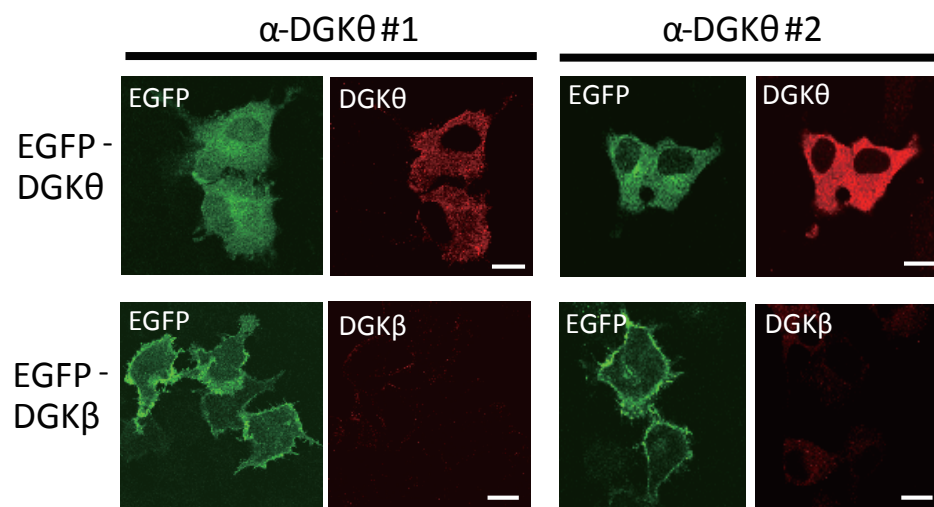

Sup. Data 1. Ueda et al.

Supplement: Additional file 2: Figure S2 — Specificity of the anti-DGKθ antibody against DGKθ and other DGKs. EGFP-fused DGKθ [45] and DGKβ [13] were transiently expressed in HeLa cells and cultured for 24 hr. The cells were fixed with 4% paraformaldehyde in PBS (-) and permeabilized with 0.3% Triton X -100 in PBS (-). Immunofluorescence staining was performed using the indicated antibodies followed by usual method [46]. Scale bar, 20 μm. [file 1471-213X-13-35-S2.pdf]

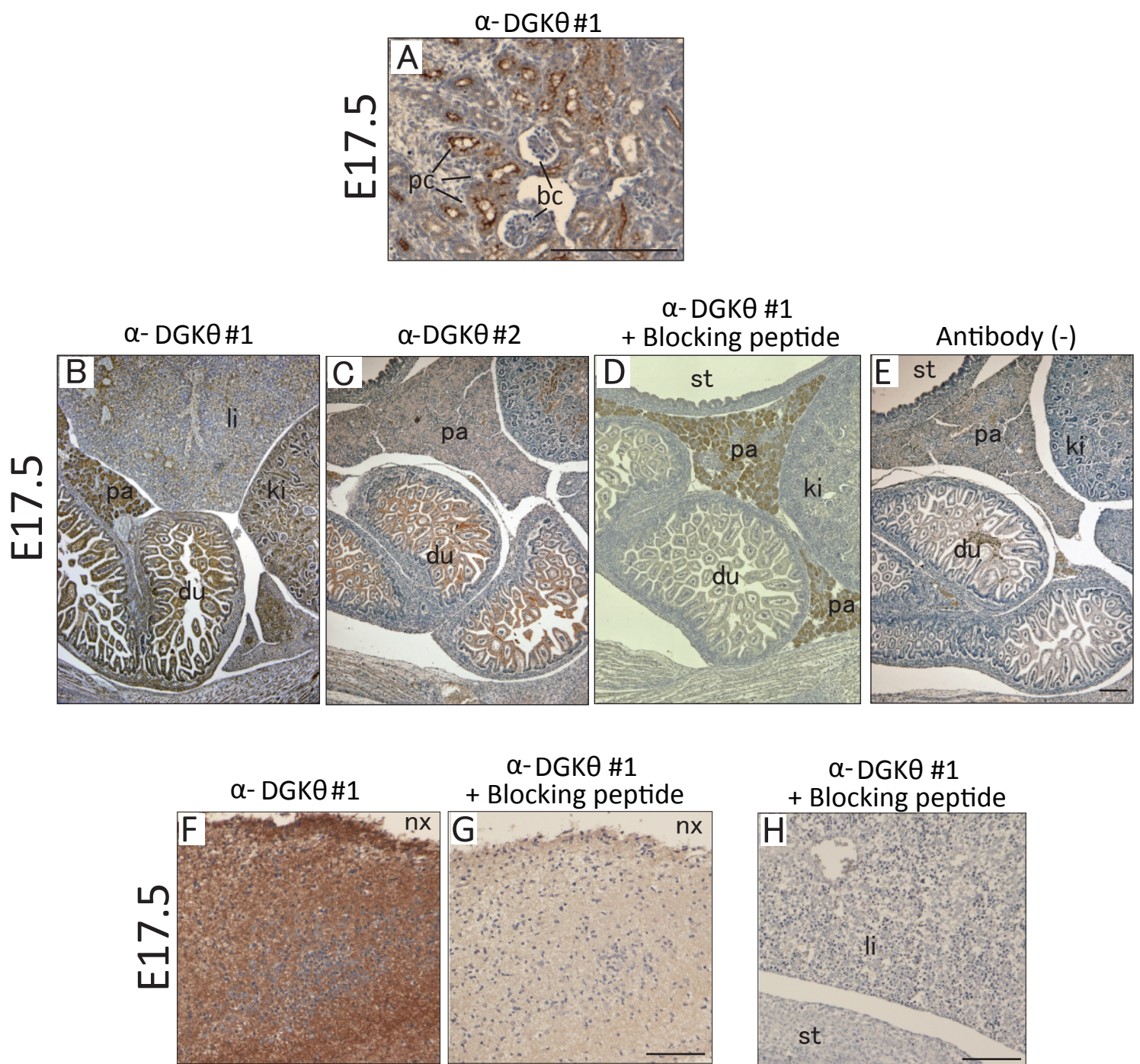

Figure S2. Ueda et al.

Supplement: Additional file 3: Figure S3 — Expression patterns of DGKθ at E17.5. (A) High-magnification images showing the area of the renal medulla at E17.5. Collecting tubule (ct), Bowman’s capsule (bc). (B-H) Sagittal sections showing the staining pattern of anti-DGKθ antibody in abdomen and head area. Stomach (st), pancreas (pa), kidney (ki), duodenum (du), liver (li), neocortex (nx). [file 1471-213X-13-35-S3.pdf]
